# 1198. More is not Merrier: Mitigating Meropenem Misuse in the ICU at an Academic Medical Center

**DOI:** 10.1093/ofid/ofad500.1038

**Published:** 2023-11-27

**Authors:** Angela Huang, Alicia Sacco, Aaron Skolnik, Eric Siebeneck, Dan Ilges, Maria T Seville

**Affiliations:** Mayo Clinic Arizona, Phoenix, Arizona; Mayo Clinic Arizona, Phoenix, Arizona; Mayo Clinic Arizona, Phoenix, Arizona; Mayo Clinic Arizona, Phoenix, Arizona; Mayo Clinic Arizona, Phoenix, Arizona; Mayo Clinic Arizona, Phoenix, Arizona

## Abstract

**Background:**

Inappropriate use of carbapenems is a major driver of antimicrobial resistance. The intensive care unit (ICU) at Mayo Clinic Arizona had a higher meropenem (MER) days of therapy (DOT) per 1000 patient days (PD) compared to other Mayo Clinic Enterprise sites, despite low rates of Pseudomonal resistance to narrower spectrum anti-pseudomonal beta-lactams.

**Methods:**

A multidisciplinary team of infectious diseases (ID) and critical care medicine (CCM) physicians and pharmacists was created to reduce inappropriate use of MER in the ICU. The baseline rate of appropriateness was determined by evaluating all MER days in the ICU during Q4 2019 and Q4 2020. Each MER day was designated as appropriate or inappropriate based on pre-determined criteria after review by at least one ID and one CCM member. Discordant days were evaluated by the whole team to achieve consensus. The team provided education to the CCM department on the initiative, baseline data, and strategies to reduce inappropriate MER use. The active intervention included prospective audit and feedback of all patients on MER performed by the ID clinical pharmacists Mon-Fri 0700-1530 in Q4 2022. DOT/1000 PD was assessed for the pre-intervention and intervention periods.

**Results:**

The baseline rate of inappropriate MER use in the ICU was 52% (442/846 days). The two most common reasons for inappropriate MER use in the baseline period were: susceptible organism for which a narrower agent could have been used (93/442, 21%), and infectious workup/cultures negative (89/442, 20%). During the intervention period, the inappropriate rate of MER use was reduced to 23%, below the pre-determined goal of 30%. The MER DOT/1000 PD in the baseline period were 103.9 (Q4 2019) and 151.5 (Q4 2020) versus 66.1 (Q4 2022) in the intervention period. There was no difference in ICU sepsis-related mortality in the pre-intervention vs intervention period (13.4 vs 0%, p=0.04).

**Conclusion:**

A multidisciplinary team approach, including education and prospective audit and feedback, reduced inappropriate MER use in the ICU of an academic medical center from 52 to 23%. An associated decrease in MER DOT/1000 patient days was also observed.

**Disclosures:**

**All Authors**: No reported disclosures

